# Optimising access: safety and efficacy of venous closure devices in cryoballoon pulmonary vein isolation

**DOI:** 10.3389/fcvm.2025.1704394

**Published:** 2025-11-18

**Authors:** S. Hatahet, C. H. Heeger, S. S. Popescu, M. Delgado, H. Grasshoff, A. Traub, S. Reincke, B. Subin, K. Ukita, K. Klotz, M. Küchler, J. Vogler, C. Eitel, J. Nikorowitsch, J. P. Wenzel, K. H. Kuck, J. Meyer-Waeterling, R. R. Tilz

**Affiliations:** 1 Department of Rhythmology, University Heart Center Lübeck, University Hospital Schleswig-Holstein, Lübeck, Germany; 2 German Center for Cardiovascular Research (DZHK), Partner Site Hamburg/Kiel/Lübeck, Lübeck, Germany; 3 Department of Rhythmology, Cardiology and Internal Medicine, Asklepios Klinik Hamburg Altona, Hamburg, Germany; 4 Department of Rheumatology and Clinical Immunology, University Hospital Schleswig-Holstein, Lübeck, Germany; 5 Department of Anesthesiology and Intensive Care Unit, University Hospital Schleswig-Holstein, Lübeck, Germany; 6 Department of Cardiology and Intensive Care Unit, Asklepios Klinik Hamburg St. Georg, Hamburg, Germany

**Keywords:** venous closure device, atrial fibrillation, pulmonary vein isolation, cryoballoon ablation, venous access

## Abstract

**Background/objectives:**

Despite technological progress in atrial fibrillation (AF) ablation, vascular access complications remain common. Venous closure systems (VCS) may reduce these events and improve patient comfort, but data on their safety and efficacy following cryoballoon-based pulmonary vein isolation (CB-PVI) are limited. This study assessed acute and long-term outcomes of VCS vs. manual compression and figure-of-eight suture after CB-PVI.

**Methods:**

We conducted a prospective, single-centre observational study comparing VCS with figure-of-eight suture plus manual compression post-CB-PVI. VCS patients who underwent CB-PVI between September 2022 and August 2023 were enrolled; controls were a 1:1 age-, sex-, and anticoagulation-matched cohort treated between January 2016 and May 2021. Ultrasound-guided access was used in all VCS cases and routinely from 2018 in controls. Pressure bandage time was ≥60 min in VCS vs. ≥4 h in controls. Vascular complications, emergency department (ED) visits, and readmissions were assessed over 12 months.

**Results:**

A total of 280 patients were included (mean age 70; 46.4% female; 38.9% paroxysmal AF). The VCS group had higher rates of hypertension (*p* = 0.036), coronary disease (*p* = 0.026), and body mass index (BMI) (*p* = 0.006). Groin-related periprocedural complications were similar (22.9% vs. 22.1%, *p* = 0.886); all were minor in the VCS group. One major complication occurred in controls. No groin-related ED visits occurred in the VCS group; one occurred in controls. Thirty-day ED visits were lower with VCS (3.6% vs. 15.1%, *p* < 0.001). Follow-up showed a trend toward fewer complications (2.5% vs. 8.5%, *p* = 0.053). Subgroup analysis (ultrasound-guidance only) confirmed these findings.

**Conclusion:**

VCS following CB-PVI is safe and feasible. No significant difference regarding acute, mid-term, and long-term groin complications was observed.

## Graphical Abstract

**Figure:**
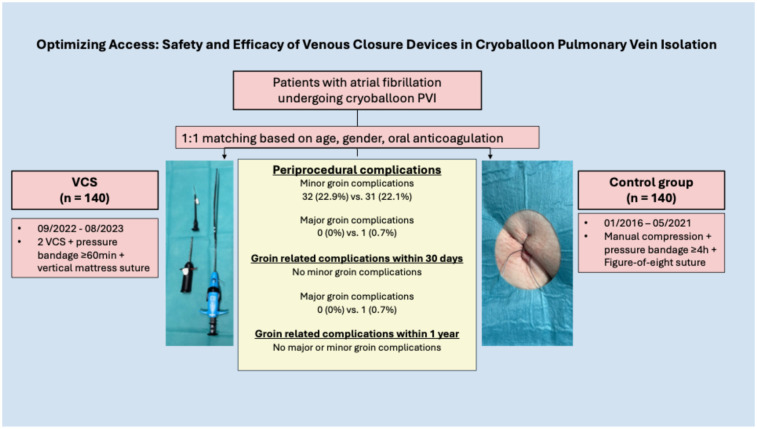

## Introduction

Pulmonary vein isolation (PVI) remains the cornerstone therapy for atrial fibrillation (AF) (1). Recent technological advances have led to a steeper learning curve and shorter procedure times. Single-shot PVI techniques, such as cryoballoon (CB)-based PVI, have proven to be both safe and effective (2). However, vascular access site complications remain the most common adverse events, with reported incidences ranging from 1.8% to 4.0%, contributing to increased morbidity and delayed hospital discharge (3–6).

As a result, improving vascular access site safety has become a growing focus of clinical research.

In the context of same-day discharge, effective vascular access management—aimed at minimising bleeding and enabling early ambulation—is essential for the success of ambulatory care strategies. In addition to the use of smaller sheaths and fewer puncture sites, common approaches to reduce access-related complications include ultrasound-guided puncture, manual compression, and the figure-of-eight suture technique (7–10). Venous closure systems (VCS) may offer further improvements in vascular access management. Devices such as the Perclose ProStyle™ and Perclose ProGlide™ (Abbott Vascular, CA, USA) and the VASCADE MVP® (Haemonetics Corporation, MA, USA) have been shown to be safe and effective alternatives to manual compression following PVI (5, 11–14). In particular, the use of VCS following CB- and pulsed-field ablation-based PVI has demonstrated reduced time to hemostasis and ambulation when compared with manual compression and the figure-of-eight suture technique. In addition, shorter time to ambulation associated with VCS use may translate into improved patient comfort and overall procedural satisfaction (5, 14). However, no data are currently available comparing VCS directly with conventional hemostasis strategies—manual compression and figure-of-eight suture—exclusively following CB-based PVI. This single-centre, observational study aims to compare VCS with conventional hemostasis methods following CB-based PVI under real-world conditions over a 12-month follow-up (FU) period.

## Methods

### Patient population

All consecutive patients with symptomatic AF who underwent *de novo* cryoballoon-based pulmonary vein isolation (CB-PVI) with VCS between September 2022 and August 2023 were prospectively enrolled in the Lübeck Ablation Registry. For comparison, a 1:1 matched control cohort—drawn from the institutional ablation registry and treated between January 2016 and May 2021—was identified based on age, sex, and oral anticoagulation status. In the control group, conventional hemostasis techniques, including manual compression and figure-of-eight suture, were used. A subset of VCS-treated patients (24.3%) also participated in the STYLE-AF study.

The study was approved by the local ethics committee (Lübeck Ablation Registry, ethical review board number: 2024-377_1) and conducted in accordance with the ethical standards of the 1964 Declaration of Helsinki and its later amendments (15). All patients provided written informed consent for the procedure and were enrolled in the Lübeck Ablation Registry.

### Preprocedural management

Preprocedural transoesophageal echocardiography was performed to exclude intracardiac thrombi in patients who had not received uninterrupted therapeutic dosing of direct oral anticoagulants (DOACs) for at least 3 weeks (16, 17). For patients on vitamin K antagonists, a periprocedural international normalised ratio (INR) of 2.0–3.0 was targeted. In patients receiving DOACs, the morning dose was withheld on the day of the procedure.

### Intraprocedural management

All CB-PVI procedures were performed in accordance with the institutional standard protocol. Detailed intraprocedural management has been described previously (5, 18–20).

Procedures were conducted under deep sedation using a combination of propofol, midazolam, and fentanyl. Vascular access was obtained via two punctures of the right femoral vein. In the VCS group, all punctures were performed under ultrasound guidance, whereas in the control group, ultrasound guidance was routinely implemented from 2018 onward.

A single transseptal puncture was performed under fluoroscopic guidance using a standard Brockenbrough needle and an 8.5 F transseptal sheath (SL1, Abbott, Illinois, USA) to access the left atrium. Following transseptal access, an intravenous bolus of heparin was administered to maintain an activated clotting time (ACT) > 300 s throughout the procedure. PVI was performed using either the POLARx™ system (Boston Scientific, St. Paul, MN, USA) or the second-/fourth-generation Arctic Front CB (Medtronic, Inc., Minneapolis, MN, USA). The associated steerable sheaths had outer diameters of 15.9 F (POLARSHEATH, Boston Scientific) and 15 F (FlexCath Advance™, Medtronic). A spiral mapping catheter (Achieve™, Medtronic, Inc., or POLARMAP™, Boston Scientific) was positioned in the target pulmonary vein to guide and monitor isolation. The administration of protamine at the conclusion of the procedure was permitted in accordance with institutional protocols and was at the discretion of the operator.

#### VCS group

**Figure 1:**
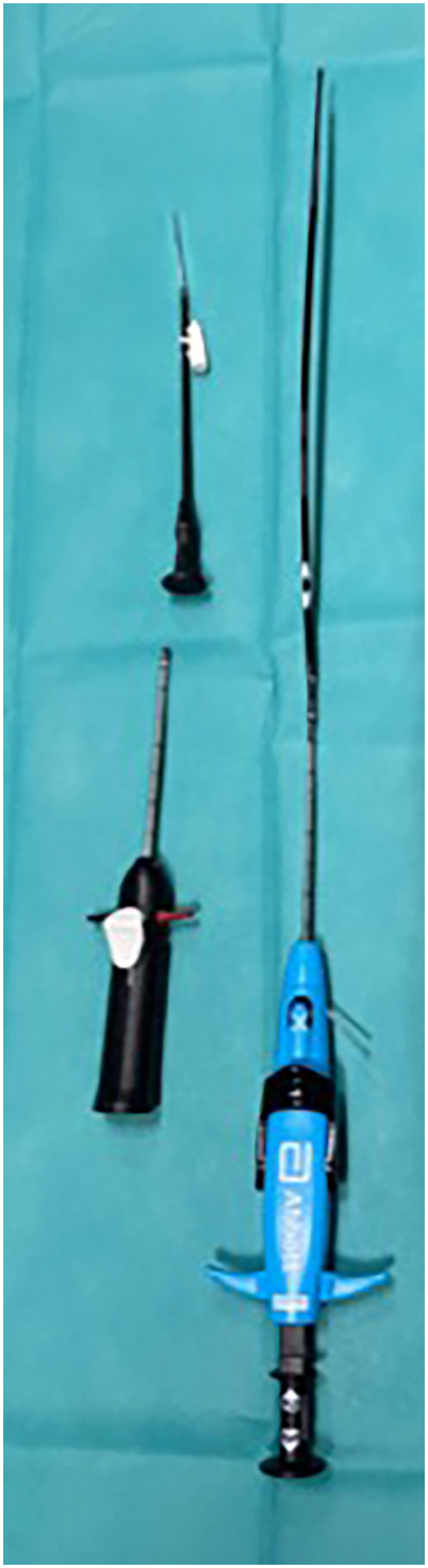
Venous closure device.

After a successful ultrasound-guided venous puncture, a guidewire was inserted, and an 8 F introducer sheath was temporarily advanced. The sheath was then removed, and the first VCS (ProGlide™ or ProStyle™) was advanced over the wire and deployed according to the manufacturer's instructions. Subsequently, the guidewire and the 8 F sheath were reintroduced to maintain access. Following this, the sheath was again removed, and a second VCS was inserted over the wire and deployed at an angle of approximately 30°–45° relative to the first. Subsequently, the guidewire and the 8 F sheath were reintroduced to continue with the procedure as per standard workflow. At the end of the procedure, following final sheath removal and activation of the suture-mediated closure, manual compression was applied if deemed necessary by the operator. To optimise skin adaptation and reduce superficial bruising, a vertical mattress suture (Donati technique) was placed at each access site (Figure 1). A pressure bandage was applied thereafter.

#### Control group

**Figure 2:**
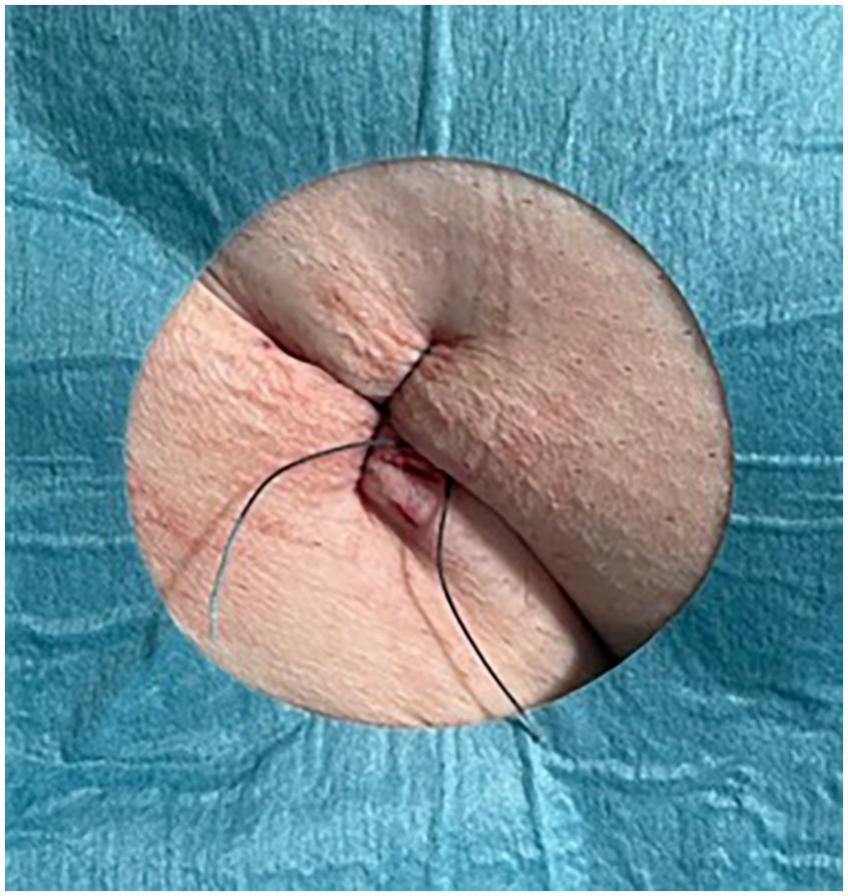
Figure-of-eight suture.

After sheath removal, a figure-of-eight suture and manual compression were applied. A pressure bandage was placed once hemostasis was achieved (Figure 2). The duration of compression was adjusted according to the individual clinical scenario.

### Postprocedural management

Pericardial effusion was ruled out at the end of the procedure, 1 h post-intervention, and again on the following day. The pressure bandage was removed after a minimum of 60 min in the VCS group and after at least 4 h in the control group. Both the vertical mattress suture and the figure-of-eight suture were removed on the day after ablation.

All patients underwent continuous non-invasive monitoring of blood pressure, oxygen saturation, and electrocardiogram (ECG), and received a 24 h Holter ECG. Oral anticoagulation was resumed 6 h after the procedure and continued for at least 3 months, with further continuation based on the CHA_2_DS_2_-VASc score, in accordance with the prevailing ESC guidelines (16, 17). Antiarrhythmic drugs were prescribed for 3 months during the blanking period.

### Follow-up

Outpatient follow-up visits were recommended at 3, 6, and 12 months after the ablation procedure, either in our institution's outpatient department or with the referring cardiologist. Follow-up assessments included a review of clinical history, a 12-lead ECG, and a 24 h Holter ECG. Atrial arrhythmia recurrence was defined as the occurrence of any atrial arrhythmia beyond the blanking period.

At the end of the follow-up period, the hospital information system was reviewed to identify potential complications and adverse events. Vascular access site complications were categorised as either minor or major. A complication was classified as major if it resulted in permanent harm, required surgical or interventional treatment, involved bleeding necessitating transfusion, prolonged hospitalisation of >48 h, or led to death (21). Complications not fulfilling these criteria were considered minor. Haematomas were classified as small (<6 cm) or large (>6 cm) based on clinical examination.

### Subgroup analysis

Ultrasound-guided venous puncture was introduced as the standard approach at our institution in 2018. Consequently, not all patients in the control group underwent ultrasound-guided access. To account for the potential influence of ultrasound guidance on outcomes, a subgroup analysis was performed comparing the VCS group to only those control patients who received ultrasound-guided puncture.

### Statistical analysis

Continuous data were initially analysed by the Shapiro–Wilk test for normal distribution. They are described as the mean ± standard deviation (SD) for normally or as median and interquartile range [median (quartile 1, quartile 3)] for non-normally distributed data. Student's *t*-test was used for comparing the mean value of a variable between two study populations. In case of non-normal distribution, the Mann–Whitney *U* test was used. Categorial variables were shown by absolute (*n*) and relative (%) frequencies. They were compared by the usage of Fisher's exact test or chi-squared test depending on sample size. Event-free survival was estimated with the Kaplan–Meier method and compared via the log-rank test. Data matching was made by the use SPSS version 29.0 (IBM SPSS Statistics), and all the following calculations were made by Jamovi version 2.6.25. All *p*-values are two-sided, and *p* < 0.05 was considered significant.

## Results

### Study population

A total of 140 consecutive patients were analysed (mean age 70 years; 46.4% female; 56.4% with persistent AF). Arterial hypertension (81.0% vs. 70.0%, *p* = 0.036), vascular disease (50.7% vs. 32.9%, *p* = 0.002), and coronary artery disease (35.7% vs. 23.6%, *p* = 0.026) were more prevalent in the VCS group. Patients treated with VCS also had a significantly higher body mass index (BMI) (*p* = 0.006). Detailed baseline characteristics are summarised in Table 1.

**Table 1:** Patient characteristics.

Variables	Total cohort (*n* = 280)	VCS (*n* = 140)	Control (*n* = 140)	*p-*value
Demographic data				
Age (years), median (UQ; OQ)	70 (62.8; 77)	70.5 (63; 77)	70 (62; 77)	0.949
Female gender, *n* (%)	130 (46.4)	65 (46.4)	65 (46.4)	1
BMI (kg/m^2^), median (UQ; OQ)	27.7 (24.5; 31)	28.3 (25.3; 31.6)	26.6 (24.1; 30.2)	**0**.**006**
Atrial fibrillation				
Paroxysmal, *n* (%)	109 (38.9)	55 (39.3)	54 (38.6)	0.598
Persistent, *n* (%)	162 (57.9)	79 (56.4)	83 (59.3)	
Long persistent, *n* (%)	9 (3.2)	6 (4.3)	3 (2.1)	
Comorbidities				
Arterial hypertension, *n* (%)	213 (76.1)	114 (81)	99 (70)	**0**.**036**
Diabetes, *n* (%)	28 (10)	18 (12.9)	10 (7.1)	0.111
Vascular disease, *n* (%)	119 (42.5)	71 (50.7)	48 (34.3)	**0**.**005**
Coronary heart disease, *n* (%)	83 (29.6)	50 (35.7)	33 (23.6)	**0**.**026**
Systolic heart failure, *n* (%)	32 (11.4)	14 (10)	18 (12.9)	0.452
Cardiomyopathy, *n* (%)	30 (10.7)	14 (10)	16 (11.4)	0.699
Stroke/TIA, *n* (%)	26 (9.3)	9 (6.4)	17 (12.1)	0.100
Bleeding, *n* (%)	8 (2.9)	3 (2.1)	5 (3.6)	0.723
Echocardiography				
LVEF (%), median (UQ; OQ)	55 (50; 56)	55 (50; 60)	55 (53; 55)	0.341
*Number*, *n*	*191*	*66*	*125*	
Medication				
Oral anticoagulation, *n* (%)	258 (92.1)	129 (92.1)	129 (92.1)	1
Antiarrhythmic drugs	59 (21.1)	23 (16.4)	36 (25.7)	0.057
Scores				
NYHA				
*Number*, *n*	*173*	*50*	*123*	
Median (UQ; OQ)	1 (1; 2)	2 (1; 2)	1 (1; 2)	**<0**.**001**
EHRA				
Median (UQ; OQ)	2 (2; 2.25)	2 (2; 2)	2 (2; 3)	**0**.**011**
CHA_2_DS_2_-VASc score				
Median (UQ; OQ)	2 (1; 4)	3 (1; 4)	2 (1; 4)	0.248
HASBLED score				
Median (UQ; OQ)	2 (1; 2)	2 (1; 2)	2 (1; 2)	0.659

TIA, transitory ischemic attack; LVEF, left ventricular ejection fraction; NYHA, New York Heart Association.

The bold values indicate statistically significant *p*-values.

### Periprocedural data

Ultrasound-guided venous access was performed significantly more often in the VCS group (100% vs. 68.8%, *p* < 0.001). The most frequently used closure device in the VCS group was ProGlide™ (82.1%), followed by ProStyle™ (17.9%). VCS failure occurred in 2.9% of patients, who were subsequently managed with a figure-of-eight suture and manual compression to achieve hemostasis.

Fluoroscopy time [9.15 (6.57; 12.1) vs. 11.3 (7.50; 16.4) min, *p* < 0.001] and total procedure time [52 (44.8; 60) vs. 55 (45; 70) min, *p* = 0.047] were significantly shorter in the VCS group compared with the control group.

The most used CB was the fourth-generation Arctic Front Advance Pro (52.1%), followed by POLARx™ (28.6%) and the second-generation Arctic Front (19.3%).

**Figure 3:**
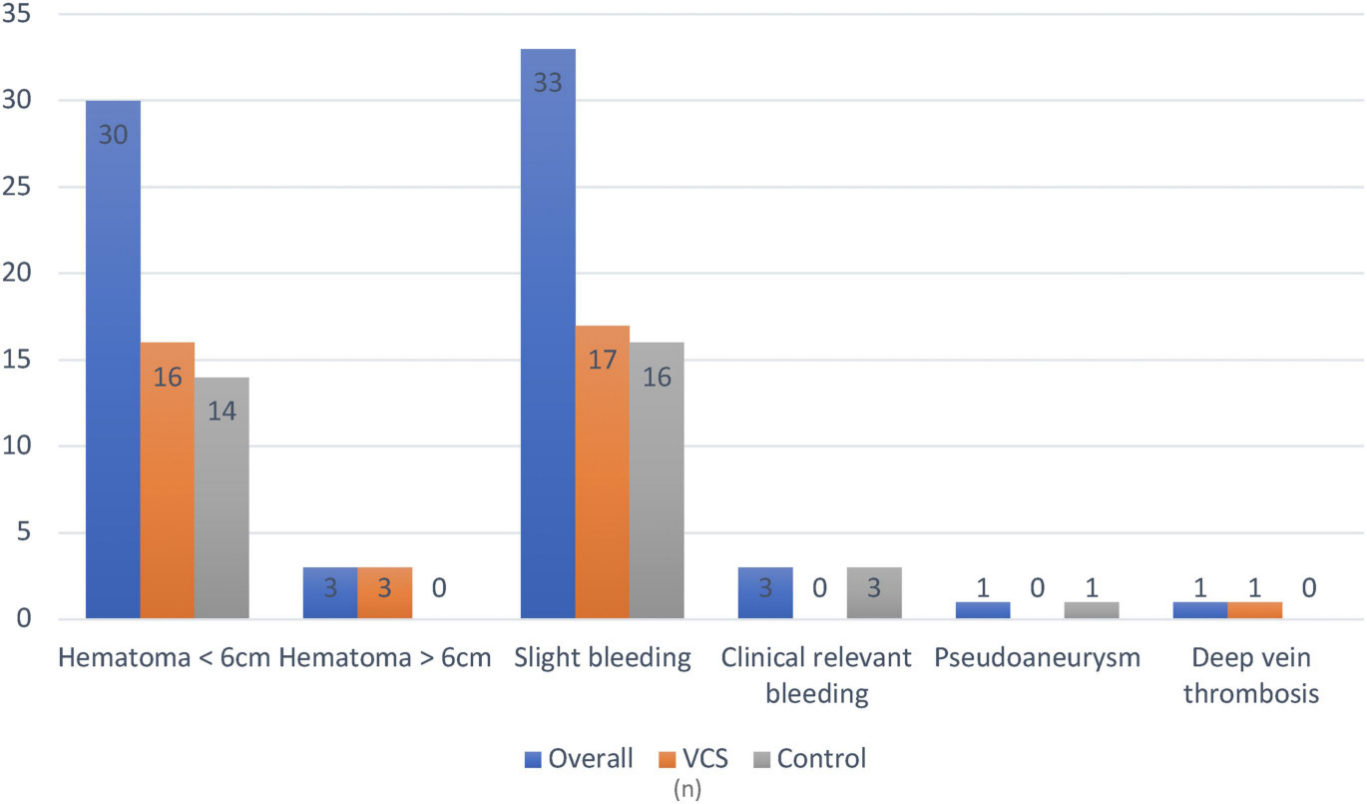
Overview of periprocedural groin complications. VCS, venous closure system.

Overall, vascular access site complications occurred in 22.5% of all patients. No major complications were reported in the VCS group, whereas one major complication (pseudoaneurysm) occurred in the control group (*p* = 1). Clinically relevant bleeding events not requiring transfusion were observed in 2.1% of control group patients and none in the VCS group. The most frequent complication in both groups was groin haematoma <6 cm (11.4% vs. 10.0%, *p* = 0.699). One patient in the control group experienced a femoral pseudoaneurysm that required surgery. The postoperative course was complicated by sepsis and multiorgan failure, resulting in death. Same-day discharge was performed exclusively in the VCS group (7.9% vs. 0%, *p* < 0.001). Detailed results are presented in Tables 2, 3 and Figure 3.

**Table 2:** Periprocedural data.

Variables	Total cohort (*n* = 280)	VCS (*n* = 140)	Control (*n* = 140)	*p-*value
Procedural data				
Isolation of all PV, *n* (%)	278 (99.3)	140 (100)	138 (98.6)	0.498
CB, *n* (%)				
POLARx™	80 (28.6)	65 (46.4)	15 (10.7)	**<0**.**001**
Arctic Front 2. generation	54 (19.3)	0 (0)	54 (38.6)	
Arctic Front 4. generation	146 (52.1)	75 (53.6)	71 (50.7)	
Fluoroscopy time (min), median (UQ; OQ)	10 (7.1; 14.2)	9.15 (6.57; 12.1)	11.3 (7.50; 16.4)	**<0**.**001**
Dose area product (cGycm²), median (UQ; OQ)	920 (463; 1,866)	855 (472; 1,408)	1,140 (423; 2,380)	0.097
Procedural time (min), median (UQ; OQ)	53 (45; 62.3)	52 (44.8; 60)	55 (45; 70)	**0**.**047**
Contrast agent (ml), median (UQ; OQ)	70 (45; 62.3)	70 (60; 80)	60 (60;100)	0.404
Groin-related procedural data				
Ultrasound-guided puncture, *n* (%)	236 (84.3)	140 (100)	96 (68.8)	**<0**.**001**
VCS				
Amount VCS, *n* (%)				
0	140 (50)	0	140 (100)	**<0**.**001**
1	0 (0)	0	0	
2	137 (48.9)	137 (97.9)	0	
3	3 (1.1)	3 (2.1)	0	
Type VCS, *n* (%)				
No VCS	140 (50)	0 (0)	140 (100)	**<0**.**001**
ProGlide™	115 (41.1)	115 (82.1)	0	
ProStyle™	25 (8.9)	25 (17.9)	0	
VCS failure, *n* (%)		4 (2.9)		
Periprocedural complications without groin complications				
Patients with complications in total, *n* (%)	28 (10)	15 (10.7)	13 (9.3)	0.690
Pericardial effusion during ablation, *n* (%)				
Yes, with intervention	1 (0.4)	0 (0)	1 (0.7)	1
Yes, without intervention	1 (0.4)	0 (0)	1 (0.7)	1
Pericardial effusion following ablation, *n* (%)				
Yes, with intervention	2 (0.7)	0 (0)	2 (1.4)	0.498
Yes, without intervention	2 (0.7)	0 (0)	2 (1.4)	0.498
Stroke/TIA, *n* (%)	1 (0.4)	0 (0)	1 (0.7)	1
Phrenic nerve palsy, *n* (%)				
Transient	1 (0.4)	0 (0)	1 (0.7)	1
Until the end of the procedure	13 (4.6)	7 (5)	6 (4.3)	1
AV-Block III° with intervention, *n* (%)	1 (0.4)	0 (0)	1 (0.7)	1
Pulmonary artery embolism, *n* (%)	1 (0.4)	0 (0)	1 (0.7)	1
Pneumonia, *n* (%)	1 (0.4)	1 (0.7)	0 (0)	1
Other complications, *n* (%)	13 (4.6)	7 (5)	6 (4.3)	1
Death, *n* (%)	1 (0.4)	0 (0)	1 (0.7)	1

The bold values indicate statistically significant *p*-values.

**Table 3:** Groin-related periprocedural complications. Since some patients experienced more than one type of complication, the total number of complications exceeds the number of individuals affected.

Variables	Total cohort (*n* = 280)	VCS (*n* = 140)	Control (*n* = 140)	*p-*value
Overall patients with groin complications, *n* (%)	63 (22.5)	32 (22.9)	31 (22.1)	0.886
Major, *n* (%)	1 (1.6)	0 (0)	1 (3.2)	0.492
Minor, *n* (%)	63 (100)	32 (100)	31 (100)	1
Haematoma < 6 cm, *n* (%)	30 (10.7)	16 (11.4)	14 (10)	0.699
Haematoma > 6 cm, *n* (%)	3 (1.1)	3 (2.1)	0 (0)	0.247
Minor bleeding, *n* (%)	33 (11.8)	17 (12.1)	16 (11.4)	0.853
Clinically relevant groin bleeding, *n* (%)	3 (1.1)	0 (0)	3 (2.1)	0.247
Pseudoaneurysm, *n* (%)	1 (0.4)	0 (0)	1 (0.7)	1
Deep vein thrombosis (%)	1 (0.4)	1 (0.7)	0 (0)	1

### Short-term follow-up (<30 days)

**Figure 4:**
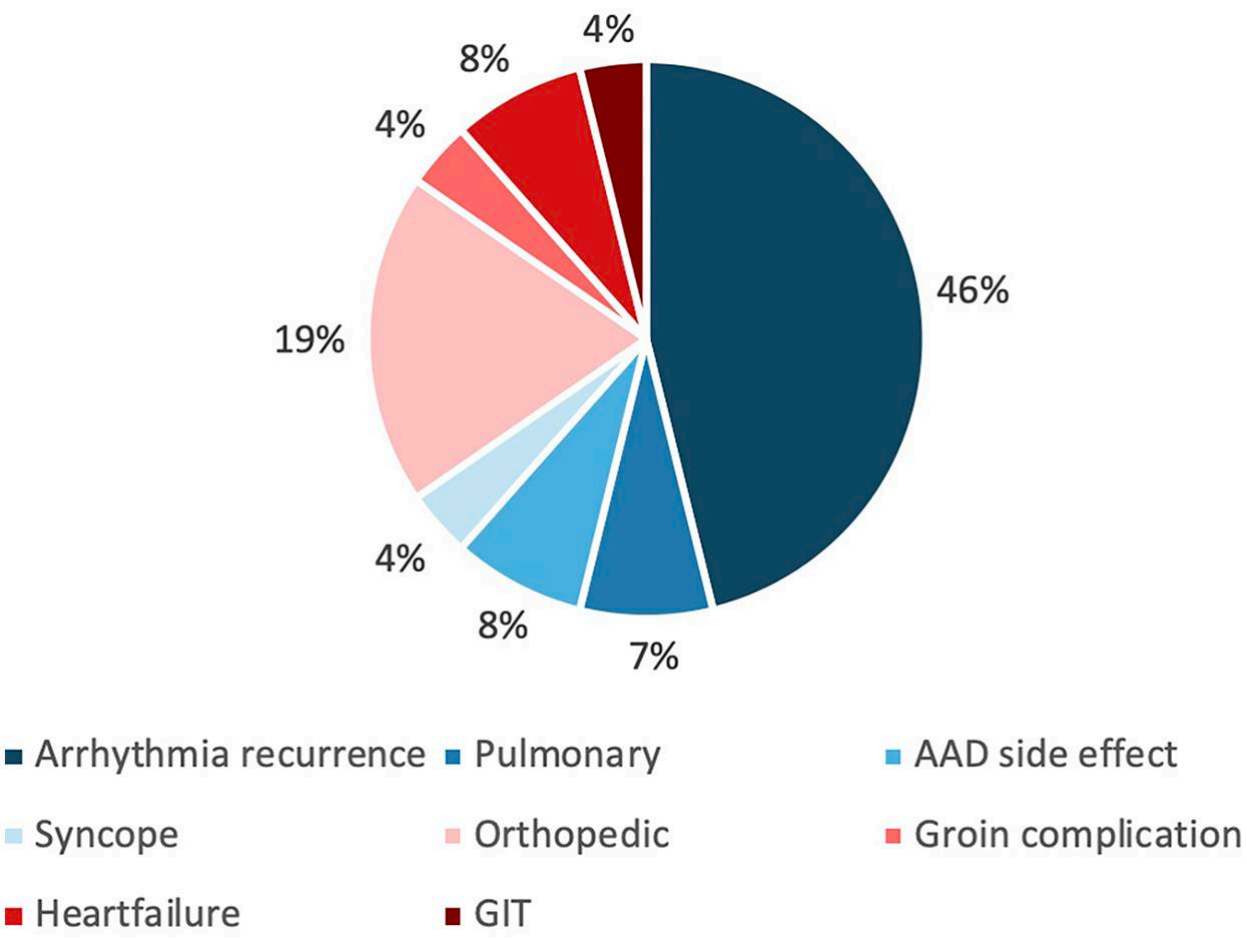
Overview of causes of emergency department visits within 30 days post-ablation. AAD, antiarrhythmic drug; GIT, gastrointestinal tract.

Within 30 days following ablation, a significantly higher number of patients in the control group presented to the emergency department (ED) (15.1% vs. 3.6%, *p* < 0.001). One patient (4.8%) in the control group presented with painful groin swelling, which was diagnosed as a pseudoaneurysm. All other ED presentations were unrelated to vascular access site complications. The most common reason for ED visits was arrhythmia recurrence (46%), followed by orthopaedic complaints (19%) (Table 4, Figure 4).

**Table 4:** Presentation in the emergency department within 30 days post-ablation.

Variables	Total cohort (*n* = 279)	VCS (*n* = 140)	Control (*n* = 139)	*p-*value
Presentation in emergency department, *n* (%)				
Total	26 (9.3)	5 (3.6)	21 (15.1)	**<0** **.** **001**
Groin-related	1 (3.8)	0 (0)	1 (4.8)	1

The bold values indicate statistically significant *p*-values.

### Long-term follow-up (>30 days)

Follow-up beyond 30 days was completed in 89.3% of patients, with a median duration of 370 days (363; 390). A significantly lower arrhythmia recurrence rate beyond the blanking period was observed in the VCS group (26.7% vs. 40.8%, *p* = 0.019). The VCS group also showed numerically fewer complications (2.5% vs. 8.5%, *p* = 0.053) and other adverse events (3.3% vs. 7.7%, *p* = 0.172). No groin-related complications were reported in either group during long-term follow-up. Stroke and sick sinus syndrome each occurred in 1.6% of patients (Table 5).

**Table 5:** Follow-up.

Variables	Total cohort (*n* = 250)	VCS (*n* = 120)	Control (*n* = 130)	*p-*value
Complications				
Overall complications, *n* (%)	14 (5.6)	3 (2.5)	11 (8.5)	0.053
Death, *n* (%)	1 (0.4)	0 (0)	1 (0.8)	1
Stroke/TIA, *n* (%)	4 (1.6)	1 (0.8)	3 (2.3)	0.623
Groin complications	0 (0)	0 (0)	0 (0)	1
Other complications, *n* (%)	9 (3.6)	2 (1.7)	7 (5.4)	0.175
Sick sinus syndrome, *n* (%)	4 (44.4)	2 (100)	2 (28.6)	
ASD, *n* (%)	3 (33.3)	0 (0)	3 (42.9)	
Acute heart failure due to arrhythmia recurrence, *n* (%)	2 (22.2)	0 (0	2 (28.6)	
Other adverse events, *n* (%)	14 (5.6)	4 (3.3)	10 (7.7)	0.172
GI bleeding, *n* (%)	4 (28.6)	2 (50)	2 (20)	
Intracardiac thrombus, *n* (%)	2 (14.3)	1 (25)	1 (10)	
Myocardial infarction, *n* (%)	3 (21.4)	1 (25)	2 (20)	
Pneumonia, *n* (%)	2 (14.3)	0 (0)	2 (20)	
Pulmonary oedema, *n* (%)	1 (7.1)	0 (0)	1 (10)	
Non-procedure-related pericardial effusion, *n* (%)	2 (14.3)	0 (0)	2 (20)	

ASD, atrium septum defect; GI, gastrointestinal.

### Subgroup analysis

As previously mentioned, ultrasound-guided venous access was performed significantly more often in the VCS group (*p* < 0.001). Given this difference, a subgroup analysis was conducted excluding patients without ultrasound-guided access, resulting in 140 patients in the VCS group and 95 in the control group. The results of this analysis regarding periprocedural complications and ED visits within 30 days post-ablation were consistent with those of the overall cohort (Tables 6, 7).

**Table 6:** Subgroup analysis—groin-related periprocedural complications.

Variables	Total cohort (*n* = 236)	VCS (*n* = 140)	Control (*n* = 96)	*p-*value
Overall patients with groin complications, *n* (%)	49 (20.8)	32 (22.9)	17 (17.7)	0.338
Major, *n* (%)	1 (2)	0 (0)	1 (5.9)	0.347
Minor, *n* (%)	49 (100)	32 (100)	17 (100)	1
Haematoma < 6 cm, *n* (%)	25 (10.6)	16 (11.4)	9 (9.4)	0.615
Haematoma > 6 cm, *n* (%)	3 (1.3)	3 (2.1)	0 (0)	0.273
Minor bleeding, *n* (%)	24 (10.2)	17 (12.1)	7 (7.3)	0.226
Clinically relevant groin bleeding, *n* (%)	1 (0.4)	0 (0)	1 (1)	0.407
Pseudoaneurysm, *n* (%)	1 (0.4)	0 (0)	1 (1)	0.407
Deep vein thrombosis (%)	1 (0.4)	1 (0.7)	0 (0)	1

**Table 7:** Subgroup analysis—presentation in the emergency department within 30 days post-ablation.

Variables	Total cohort (*n* = 235)	VCS (*n* = 140)	Control (*n* = 95)	*p-*value
Presentation in emergency department, *n* (%)				
Total	21 (8.9)	5 (3.6)	16 (16.8)	**<0** **.** **001**
Groin-related	1 (3.8)	0 (0)	1 (4.8)	1

The bold values indicate statistically significant *p*-values.

## Discussion

The recently published STYLE-AF trial from our institution demonstrated the safety and feasibility of VCS in single-shot-based PVI, along with increased patient satisfaction due to shorter time to ambulation (5). However, no dedicated analysis of VCS exclusively in the setting of CB-based PVI, including long-term follow-up, has been conducted to date. The present study aims to evaluate the safety and efficacy of VCS specifically in CB-based PVI procedures.

The main findings are:

1. No significant difference regarding acute, mid-term, and long-term groin complications
2. No severe groin complications in the VCS group

Due to its short learning curve and favourable safety and efficacy profile, CB-PVI has become an established method for first-time ablation procedures (2, 22). However, vascular access site complications remain the most frequent periprocedural events (4). Prolonged manual compression, figure-of-eight suture techniques, and the extended application of often uncomfortable pressure bandages have long represented the standard approach to achieve hemostasis (4, 7, 10).

The introduction of VCS offers the potential to improve both procedural safety and patient comfort (5). In our study, procedural time was significantly shorter in the VCS group. This observation may be explained by the fact that procedures in the VCS group were performed more recently, at a time when CB technology, procedural workflows, and overall operator experience had further advanced. Therefore, the shorter procedural times are not directly attributable to the use of VCS itself, but rather to the overall procedural evolution and increased experience during the study period (20).

VCS failure occurred in 2.9% of patients, which is consistent with previous reports from our centre (5). In these cases, hemostasis was successfully achieved with manual compression and a figure-of-eight suture. Despite a higher burden of comorbidities in the VCS group, no significant difference in the rate of periprocedural groin complications was observed between groups. The overall incidence of groin-related complications was comparable to previous studies (5), but importantly, no severe access site complications occurred in the VCS group. Although small groin haematomas (<6 cm) were more common in the VCS group, no clinically relevant bleeding events were reported. In contrast, such events did occur in the control group. None of the patients in either group required a blood transfusion; however, clinically significant bleeding would have precluded same-day discharge.

Same-day discharge following CB-PVI is increasingly viewed as a cornerstone of modern electrophysiological care (23, 24). In our cohort, same-day discharge was only performed in the VCS group, reflecting the more recent timing of their ablation procedures and the greater procedural efficiency and safety profile observed in this group.

Within 30 days post-ablation, ED presentations occurred more frequently in the control group. With the exception of one pseudoaneurysm case, all visits were not directly associated with vascular access site complications. The majority of presentations were due to arrhythmia recurrence or orthopaedic complaints. Orthopaedic-related ED visits were numerically more frequent in the control group (3.8% vs. 15.4%, *p* = 0.350), although this difference was not statistically significant. This may suggest an indirect association with reduced early mobility in patients treated without VCS. However, this observation remains speculative and requires confirmation in larger cohorts (5, 11).

Long-term follow-up over 12 months revealed a favourable safety profile, with numerically fewer complications and adverse events in the VCS group. Importantly, no groin-related complications were observed during the follow-up period, further supporting the long-term safety of VCS in the setting of CB-based PVI.

Given the imbalance in ultrasound-guided puncture between groups, a subgroup analysis was performed, including only patients who underwent ultrasound-guided venous access. The results of this analysis were consistent with those of the overall cohort, confirming the robustness of the findings.

## Limitations

This was a prospective, single-centre, observational study. All procedures were performed by experienced operators at a high-volume electrophysiology centre, which may limit the generalisability of the results to lower-volume centres where procedural workflows and outcomes may be affected by the learning curve. The observed differences in procedural duration might, in part, reflect a more advanced stage of the learning curve in the VCS group.

Time to ambulation, time to hemostasis, and patient comfort were not assessed systematically and should be addressed in future studies. Moreover, the unequal use of ultrasound-guided puncture between groups may have influenced the results and could have a greater impact in larger or more diverse patient populations.

## Conclusion

This study is the first to investigate VCS in CB-PVI with long-term follow-up. Despite a higher burden of comorbidities, VCS were safe and feasible, with no major access-site complications observed. Moreover, VCS-treated patients presented less frequently to the ED, which may reflect the benefits of shorter postprocedural immobilisation as demonstrated in previous studies. Overall, VCS offers patient-centred advantages without compromising safety or efficacy.

## Data Availability

The raw data supporting the conclusions of this article will be made available by the authors, without undue reservation.
